# Preventive Pharmacotherapy for Cardiovascular Disease: A Modelling Study Considering Health Gain, Costs, and Cost-Effectiveness when Stratifying by Absolute Risk

**DOI:** 10.1038/s41598-019-55372-8

**Published:** 2019-12-20

**Authors:** Nhung Nghiem, Josh Knight, Anja Mizdrak, Tony Blakely, Nick Wilson

**Affiliations:** 1 BODE3 Programme, University of Otago, Wellington, New Zealand; 2 University of Auckland, Auckland, New Zealand; 3 University of Melbourne, Melbourne, Australia

**Keywords:** Health care economics, Epidemiology, Patient education

## Abstract

Cardiovascular disease (CVD) is the leading cause of death internationally. We aimed to model the impact of CVD preventive double therapy (a statin and anti-hypertensive) by clinician-assessed absolute risk level. An established and validated multi-state life-table model for the national New Zealand (NZ) population was adapted. The new version of the model specifically considered the 60–64-year-old male population which was stratified by risk using a published NZ-specific CVD risk equation. The intervention period of treatment was for five years, but a lifetime horizon was used for measuring benefits and costs (a five-year horizon was also implemented). We found that for this group offering double therapy was highly cost-effective in all absolute risk categories (eg, NZ$1580 per QALY gained in the >20% in 5 years risk stratum; 95%UI: Dominant to NZ$3990). Even in the lowest risk stratum (≤5% risk in 5 years), the cost per QALY was only NZ$25,500 (NZ$28,200 and US$19,100 in 2018). At an individual level, the gain for those who responded to the screening offer and commenced preventive treatment ranged from 0.6 to 4.9 months of quality-adjusted life gained (or less than a month gain with a five-year horizon). Nevertheless, at the individual level, patient considerations are critical as some people may decide that this amount of average health gain does not justify taking daily medication.

## Introduction

Cardiovascular disease (CVD) is the single largest disease category causing death globally in 2017 at an estimated 17·8 million deaths, followed by neoplasms at 9·56 million deaths^1^. Furthermore, this Global Burden of Disease study noted that “the increasing prevalence of obesity might explain why death rates for cardiovascular disease are no longer declining in Australia, Austria, Brazil, Germany, Netherlands, the UK, and the USA”^1^.

Fortunately, CVD is cost-effectively preventable using such means as tobacco control but also preventive pharmacotherapy^2^. Indeed, international work has reported that statins are cost-effective for the primary prevention of CVD (eg, a Cochrane Review^3^ and a review by Kazi *et al*.^4^). A systematic review of economic evaluations in low- and middle-income countries has also reported that both lipid-lowering and blood pressure lowering medication is typically cost-effective or even cost-saving when used for primary prevention of CVD (including in combination)^2^.

Furthermore, when intensive lipid lowering treatment (relative to standard lipid lowering) is considered, it has been reported that this treatment is cost-effective in all groups (ie, in a study from the Netherlands – albeit this being for patients with established CVD^5^). Similarly, an Australian study reported that “recommending blood pressure-lowering drugs to everyone with at least 5% absolute risk and statin drugs to everyone with at least 10% absolute risk” would generate health gain and save the Australian Government $5.4 billion over the lifetime of the population (or $7.1 billion if New Zealand statin prices were matched, 2008 prices)^6^.

Nevertheless, uncertainties remain with a report that industry-funded studies of statins provide more favourable cost-effectiveness estimates^7^. Also due to country variation in disease epidemiology and costs, policy-makers need jurisdiction-specific analyses on health gain, costs and cost-effectiveness. There also needs to be better individual level information for patients on how much quality-adjusted life that they might gain from taking daily medication.

New Zealand is an ideal case study country to consider such issues, given that consideration of absolute CVD risk has been promoted to clinicians for a long time^8^, albeit with this approach still not always dominating in clinical practice^9^. There is also evidence for successful campaigns to increase preventive pharmacotherapy eg, statin use in Māori^10^, the Indigenous population. Furthermore, net health sector cost-savings from further CVD prevention are also potentially large eg, NZ$1.1 billion from a single sodium reduction intervention modelled over the remaining lifetime of New Zealand adults (n = 2.3 million 35+ year olds)^11^. Another study of the impact of tobacco tax increases in this country also estimated very large health sector cost-savings, although this model also included impacts on reducing cancers and respiratory diseases along with CVD^12^.

Given this background, we aimed to model the impact of CVD preventive pharmacotherapy by clinician-assessed absolute risk level and identify the associated health gain, impact on health system costs and cost-effectiveness for 60–64 year old males. We selected this age-group as just an initial starting point for future work on assessing this approach of considering absolute CVD risk. In addition, this age-group of men is of particular interest as it is the working age with the highest rate of CVD and improving health in this age-group may enhance productivity of the economy (including for those citizens who continue in the paid workforce after age 65 years). We focused on double therapy (a combination of a statin and an anti-hypertensive) as these preventive medicines are already widely used in this way in New Zealand and aspirin as a preventive pharmacotherapy is more controversial.

## Methods

**Figure 1:**
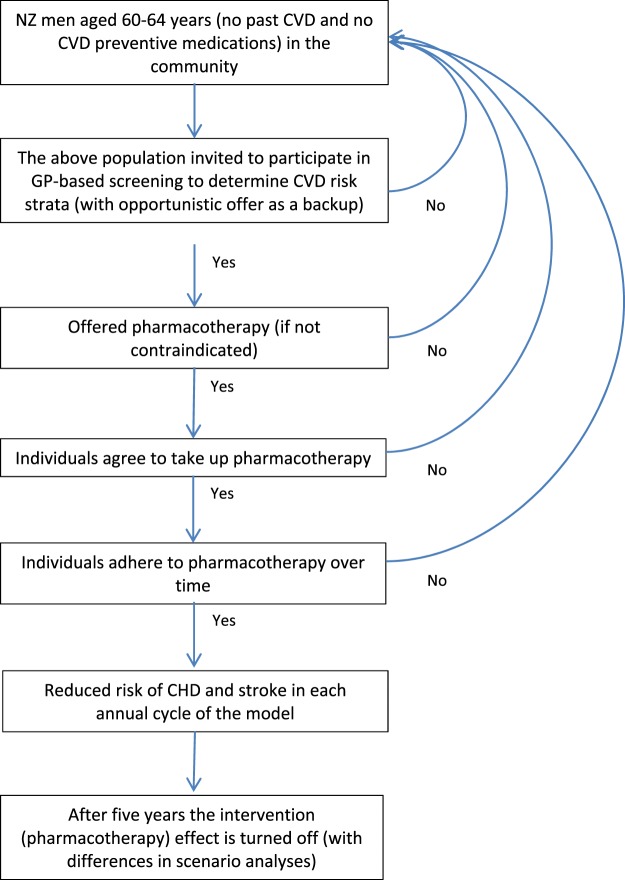
Intervention flowchart for CVD risk screening and provision of CVD preventive pharmacotherapy in this new CVD MSLT Model.

We developed a CVD multi-state life-table (MSLT) model from an established MSLT (used for tobacco control) to model the whole New Zealand population throughout their lifetime and estimated health gains and health system costs. Figure 1 provides an overview of the modelled intervention process. This is then followed by more detailed descriptions of the new CVD multi-state life-table (MSLT) model and key parameters.

### Multi-state life-table model

Our study built on the BODE^3^ Tobacco Control multi-state life-table (TC-MSLT) Model from which we have published results from previously^12–19^. This original model benefits from rich national epidemiological data by sex, age, and ethnicity (Māori and non-Māori), as well as costing data. Results from the CVD component of this model have also been validated via a head-to-head comparison with a separate model with a different structure ie, a CVD model built in TreeAge and used for dietary salt interventions^11,20,21^. The data in this TC-MSLT Model are used to estimate quality-adjusted life-years (QALYs) gained and net health system costs over the remaining life of the 2011 New Zealand population. The specific enhancements made for the current study are outlined in more detail below.

### Study population

#### Integrating CVD risk data from a synthetic national population

As the TC-MSLT Model lacked data on grouping individuals by level of absolute CVD risk, we stratified the cohorts into categories of absolute CVD risk. We utilised previous work using New Zealand-specific CVD risk prediction equations to create a synthetic simulation popoulation^22^. The variables required for the risk equation predictions were: age, sex, ethnicity, social deprivation, smoking status, diabetes status, personal history of CVD, blood pressure and lipid-lowering medication treatment, systolic blood pressure, the total cholesterol to high density lipoprotein cholesterol ratio (TC:HDL), and family history of premature CVD (with these from the PREDICT dataset, Auckland University). These risk equations were then applied to a synthetic population of 2.45 million New Zealand adults to estimate numbers and rates of CVD events. This population was formed by extracting all anonymised 30–84-year-old respondents to the 2013 census, with such variables on age, sex, ethnicity, social deprivation and smoking status. Uncertainty was generated by sampling from 100 synthetic populations. A more detailed description of this synthetic data generation is provided elsewhere^22^.

We focused on the male population aged 60–64 years who were not on CVD preventive medication (with standard deviations of the sampled means) and who had no previous diagnoses of CVD in the TC-MSLT Model. Similarly, they also had no previous diagnoses of the following: chronic kidney disease, rheumatic heart disease, congestive heart failure and atrial fibrillation^22^. Table 1 provides an example of the data for men aged 60–64 years.

**Table 1:** Example data for the predicted five-year risk of a CVD event for the synthetic national population for non-Māori and Māori men (60–64 years, with no past CVD events and on no CVD medication^22^).

Five-year cumulative absolute risk (%) strata for CVD events (fatal and non-fatal)	Non-Māori		Māori	
	Population (N)	Average risk within each risk stratum	Population (N)	Average risk within each risk stratum
>20%	56	22.9	44	22.9
>15, ≤20%	265	16.8	169	16.9
>10, ≤15%	1882	11.7	772	11.9
>5, ≤10%	19,577	6.5	2611	7.1
>0, ≤5%	36,319	3.6	875	4.1
Total	58,099	—	4470	—
Average risk*	—	5.0	—	7.9

*Calculated as the population weighted average risk across strata.

#### Building CVD risk stratification to create the CVD MSLT Model

We then took the TC-MSLT Model and modified it to create the CVD MSLT Model. This involved splitting the modelled population into three separate components (with replication for each ethnicity grouping in the age 60–64-year-group of men).

1. **Population A**: This was the group who were not on any CVD medications and did not have prevalent CVD in 2011. This group was then divided into five strata of differing levels of five-year absolute risk of a CVD event as per the proportions in the synthetic population work^22^. It is this population that was the intervention population in the model ie, those potentially offered CVD preventive pharmacotherapy.
2. **Population B**: This was the group with no prevalent CVD in 2011 but who were already on CVD preventive medication. This group were given incidence rates of CVD based on the estimated five-year absolute risk of a CVD event as per the synthetic population work^22^.
3. **Population C**: This was the group who had prevalent CVD in 2011, regardless of medication status. Again, the proportion in this group was derived from the synthetic population distribution.

Collectively these three groups cover all New Zealand men in this selected age-group. In addition, we needed to provide unique case fatality rates for the strata in Group A. There were no published New Zealand data for this (the case fatality data exist by age-group only^23^) so we considered the results from the meta-analysis by Zambon *et al*.^24^ and used the regression equation for CVD mortality by CVD incidence (Figure 2(c) in Zambon *et al*.) to mathematically disaggregate the case fatality risks by absolute risk strata – ensuring the case fatality over all strata combined was preserved.

There is also evidence that those with elevated CVD incidence also have relatively elevated non-CVD mortality rates (eg, data abstracted from the meta-analysis by Thomopoulos *et al*.^25^ albeit without age-standardisation). Based on this evidence from Thomopoulos *et al*., we specified a two-fold increase in non-CVD mortality rates for the highest compared to lowest absolute risk strata, with a linear trend over intervening risk strata in Population A (with wide uncertainty around this 2.0 value included in our modelling [confidence intervals: 1.0 to 3.0 times]).

### Model calibration

The MSLT is dynamic, meaning that our initial data from the TC-MSLT model and parameters from external literature inputs (above) once simulated through strata of CVD risk may not generate the same number of disease events, life years lived, etc, as the un-stratified population in the TC-MSLT. To be consistent with the TC-MSLT, we therefore required that the outputs summed or averaged across absolute CVD risk had to be equal to those in the non-stratified TC-MSLT at a five-year time horizon. Accordingly, we ran optimisation routines (in the R programming language) for case fatality rates (separately for CHD and stroke) and non-CVD background mortality rates (BMR). That is the weighted sum of the BMRs by absolute risk had to return the BMR for both the non-Māori and Māori populations in this age-group, ie, the BMR used in the TC-MSLT Model. This process allowed us to achieve complete matching (with the original model) of the cumulative count of CVD and non-CVD deaths after five years only. Fig. S1 in the Supplementary Material further details this process.

### Modelling approach and key parameters

For each ethnicity grouping we used the adjusted five-year CVD risk estimates (see above for an example) and ran the intervention of offering and providing (if accepted) double therapy for a five-year period. Further details on the epidemiological and intervention parameters are below and in Tables 2–4.

**Table 2:** Summary of epidemiological and cost parameters used in the modelling (see Supplementary Material Table S1 for the full details).

Input parameter/s	Source	Heterogeneity	Expected Value and 95% UI	Distribution
***Intervention parameters***				
General practitioner (GP) level screening for CVD risk: provision of offer and with the GP asking at opportunistic consultations as a backup (for both double therapy and single medications)	National District Health Board (DHB) data	Variation by Māori/non-Māori	Māori: 86% Non-Māori: 92% (using median values for all DHBs, no mean values available)	Beta, SD = ±5%
Intervention uptake by patients when recommended by a GP (for both double therapy and single medications)	NZ data^33^	No variation (see details in the column to the left)	77% overall	Normal, SD = ±10%
Decline in adherence to pharmacotherapy throughout the 5-year intervention period (for double therapy and single medications)	NZ data^34^ and authors’ assumptions	No variation (see details in the column to the left)	Over the whole 5 y period a 22.5% linear decline in adherence	Beta distribution (SD +/−5% of the cumulative reduction value)
Effect of CVD preventive pharmacotherapy on risk of CHD and stroke events	See Table 4	No variation	See Table 4	Log-normal
***Sensitivity and scenario analyses (for both double therapy and single medications)***				
Varying the discount rate	We used 0% and 6% in sensitivity analyses (as per our BODE^3^ modelling protocol^26^).			
Equity analysis	In this analysis we gave the Māori population the same potential envelope of health gain as per non-Māori, ie, the same morbidity and mortality rates as non-Māori^35^. This prevents Māori in the analysis from effectively being penalised due to poorer existing health relative to non-Māori.			
Halving of effect sizes for risk reduction (ie, treatment effect by CVD risk strata)	This scenario was considered given that the trial data might not be fully generalisable to the adult population in this target age-group (eg, trials tend to involve patients with elevated risk levels).			
5-year time horizon	As per the base-case analysis, but where the benefits (QALYs gained) and health costs were tallied up at 5 years.			
10-year time horizon	As per the base-case analysis, but where the benefits (QALYs gained) and health costs were tallied up at 10 years.			
20-year time horizon	As above but for the 20-year point.			
Continuing use of therapy for 10 years (ie, extending intervention duration in base model from 5 years to 10 years)	We assumed that after the initial 5-year decline in adherence, that adherence would then plateau (as per above in the 50% to 70% range). Of note is that for those in NZ with a known history of CVD, the use of two CVD medication categories (BP-lowering and lipid-lowering) was 70% in the older 65–74 year old age-group^36^.			
Continuing use of therapy for 20 years	As in the row above but for 20-years.			
***Costs***				
Background health system costs for all citizens (adjusted for CHD and stroke costs)	As per BODE^3^ costing methods^37^	Nil	Uncertainty: ±10% SD.	Log-normal
GP visits, prescriptions, pharmaceutical costs	See Table 3	Nil	See Table 3	See Table 3

**Table 3:** Selected additional description of intervention-specific cost parameter details (see Supplementary Material Table S2 for the full details).

Input Parameter	Source	Expected Value and 95% UI	Distri-bution
***Costs for CVD assessment and being prescribed CVD preventive pharmacotherapy***			
GP visits for initial CVD risk assessment and on-going prescriptions and check-ups (same for double therapy and single medications)	PHARMAC cost resource manual^38^	NZ$218 (in 2011 dollars) per annum over the five year intervention period	Gamma, SD ± 20%
Fasting lipid test on first consultation (required for all CVD risk assessment, both double therapy and single medications)	HealthTracker data for 2011	$28.29 (in first year of intervention period only)	Gamma, SD ± 10%
Two annual prescriptions via telephone from GP (same for double therapy and single medications)	PHARMAC cost resource manual^38^	$28.93 per annum (2011 dollars)	Gamma, SD ± 10%
Pharmacist payments for double therapy (2 medicines at 4 times year)	PHARMAC cost resource manual^38^ and pricing data for an agreement with community pharmacies^39^	$41.57 per annum (2011 dollars)	Gamma, SD ± 10%
Pharmacist payments for dispensing single medications (1 dispensing 4 times year)	As above	$20.78 per annum	Gamma, SD ± 10%
***Pharmaceuticals***			
Lipid-lowering medication (same for double therapy and single medications)	PHARMAC Online Schedule in 2017 (https://www.pharmac.govt.nz/Schedule)	$10.97 (2011 dollars)	Gamma, SD ± 10%
Anti-hypertensive (same for double therapy and single medications)	As above	$6.21 (2011 dollars)	Gamma, SD ± 10%
Total annual cost of double therapy	See above	$17.18 per annum (2011 dollars)	See for individual medicines

**Table 4:** Relative risks for preventing CVD events from preventive CVD pharmacotherapy versus no medication (95% CI) (applying to all CVD risk strata).

Outcome	Statin*	Anti-hypertensive**	Double therapy (as calculated for this study)#
Total CHD events (non-fatal and fatal)	0.73 (0.67 to 0.80) Cochrane Review^3^	0.81^40^ (0.73 to 0.89) (Using SD = 5% of the point estimate)	0.59 (0.50 to 0.69) (used in this modelling)
Total stroke events (non-fatal and fatal)	0.71 (0.62 to 0.82) USPSTF Review^29^	0.75^40^ (0.68 to 0.82) (Using SD = 5% of the point estimate)	0.53 (0.42 to 0.66) (used in this modelling)

^*^These results are consistent with a long-term trial that found that among individuals with LDL-C ≥ 190 mg/dL, pravastatin reduced the risk of CHD death, cardiovascular death and all-cause mortality by 28% (p = 0.020), 25% (p = 0.009) and 18% (p = 0.004), respectively, over a total of 20-years of follow-up^41^. USPSTF: US Preventive Services Task Force.

**Results from Law *et al*. for those aged 60–69 years for one medication at the standard dose in the range for the mean systolic BP for those in this age-group in NZ (based on NZ survey data^42^), ie, 138 mmHg for men and 132 mmHg for women.

^#^The effects from each medication are assumed to be independent. To calculate the aggregate effect of double therapy, the relative risks from each monotherapy were multiplied together. The Ersatz Excel plugin was used to generate the 95% CI by running 2000 iterations of a log-normal distribution.

The impact of the intervention in terms of health gain in QALYs were accumulated over the remainder of the cohort’s life. Of note, we assumed that the intervention period was only for five years, with treatment ending at this point for those who took up the intervention at the start (with 10 and 20 years in sensitivity analysis). Furthermore, we also assumed that CVD risks for the intervention groups all returned to average within strata risks after the five year intervention period.

Likewise, net health system costs were tallied up, including the cost of the intervention, the costs of averted health care (from preventing CVD; by including disease costs in the MSLT), and the costs of additional health care from any extended lifespan (due to health system costs assigned to all alive citizens, which with increased longevity from the intervention contribute to additional health expenditure in later life).

Sensitivity and scenario analyses were run to encompass differing discount rates (0% and 6% as per our BODE^3^ Research Protocol^26^) and the impact of taking double therapy for longer periods (10 and 20 years). Monotherapy of either a statin or an anti-hypertensive alone, were also examined.

### Ethical approval

Approval for use of anonymised administrative data as part of the BODE^3^ Programme has been granted by the Health and Disability Ethics Committees (reference number H13/049).

## Results

For men in this 60–64-year age-group, the potential offer of double therapy (a statin and an anti-hypertensive) was found to be highly cost-effective in all absolute risk strata (when using the threshold of <NZ$45,000 per QALY as being cost-effective, ie, approximately real GDP per capita in New Zealand in 2011) (Table 5). Indeed, it was extremely cost-effective in the highest risk stratum (incremental cost-effectiveness ratio (ICER): NZ$1580 per QALY gained for >20% risk) and in the lowest risk stratum (≤5%) it was still very cost-effective with an ICER of NZ$25,500 (NZ$28,200 and US$19,100 in 2018) per QALY gained (95%UI: NZ$12,300 to NZ$41,500 (NZ$13,600 to 45,900 in 2018)).

**Table 5:** Health gains (QALYs) and net health system cost impacts for 60–64-year-old men (Māori and non-Māori) from the offer of five-years of double therapy involving a statin and an anti-hypertensive, 3% discount rate, and a lifetime horizon^*^.

Five-year cumulative absolute risk strata	Total QALYs gained (non-Māori)	QALYs gained per 1000 people (non-Māori)	Total QALYs gained (Māori)	QALYs gained per 1000 people (Māori)	Total QALYs gained (ethnic groupings combined)	Net costs in NZ$ million (ethnic groupings combined)	ICER (NZ$ per QALY gained)**
>20%	16.7 (13.0 to 20.5)	289 (225 to 355)	10.2 (7.70 to 12.7)	243 (183 to 302)	26.9 (20.8 to 33.0)	$0.04 ($−0.02 to $0.1)	1580 (Dominant to $3990)
>15, ≤20%	55.4 (43.9 to 67.9)	203 (160 to 248)	28.7 (22.3 to 35.4)	177 (138 to 218)	84.0 (66.3 to 103)	$0.16 ($−0.09 to $0.39)	1930 (Dominant to $4960)
>10, ≤15%	263 (205 to 319)	135 (106 to 164)	91.7 (70.0 to 112)	124 (94.7 to 152)	354 (276 to 430)	$1.18 ($−1.5 to $2.55)	$3430 (Dominant to $7860)
>5, ≤10%	1410 (1110 to 1720)	70.0 (54.9 to 85.3)	179 (139 to 220)	71.6 (55.7 to 87.9)	1590 (1250 to 1940)	$14.8 ($4.8 to $25.3)	$9510 ($2740 to $18,000)
>0, ≤5%	1330 (1060 to 1610)	35.5 (28.3 to 43.1)	32.3 (25.6 to 39.2)	38.6 (30.6 to 46.9)	1360 (1090 to 1650)	$34.1 ($18.5 to $51.4	$25,500 ($12,300 to $41,500)

^*^For those starting with no past CVD events and no past CVD medication; using 92% screened, 77% uptake and an overall 22.5% decline in adherence over time; life-time QALYs and life-time costs but for a 5-year treatment period only, 3% discount rate, with 95% uncertainty intervals.

^**^In this context, a “Dominant” ICER means that the intervention leads to a population health gain at a net cost-saving to society, in comparison with no treatment.

The highest absolute population level health gains were not from treating men in the highest two risk categories (gaining only 26.9 and 84.0 QALYs respectively), but from the lowest two risk categories (1590 QALYs for the >5, ≤10% stratum; 1360 QALYs for the ≤5% stratum).

The per capita QALY gain for Māori men (Indigenous) was similar to non-Māori, albeit slightly greater for Māori in the lowest two risk groups (Table 5). The health gains for Māori were further increased with an “equity analysis” scenario, in which non-Māori background mortality and morbidity were used for Māori (Table S3 in the Supplementary Material). All the other sensitivity and scenario analyses produced net health gain and were cost-effective, except for the half effect size scenario, where the double therapy intervention was no longer cost-effective for the lowest risk stratum (ICERs: NZ$66,100 (NZ$73,100 in 2018), Table S3 in the Supplementary Material). As expected, the smallest health gains were seen with the base-case five-year intervention period and the largest when the intervention period was extended to either 10 or 20 years in scenario analyses (Table S4 in the Supplementary Material).

At an individual level, the health gain for those who responded to the screening offer and commenced treatment with double therapy ranged from 0.6 to 4.9 months of quality-adjusted life gained, depending on risk strata (Table 6). Slightly higher values for per capita gain were apparent when these health gains were not discounted (Table 6).

**Table 6:** Average individual level health gain associated with five-years of double therapy with (3%) and without (0%) discounting to potentially facilitate more informed patient-clinician discussions around medication use (the values at 0% discount rate are in brackets)

Five-year cumulative absolute risk strata	Quality-adjusted healthy months of life gained for the average cohort member from the offer of screening (intention-to-treat style of analysis)		Quality-adjusted healthy months of life gained for those who respond to the screening offer and commence treatment^*^	
	Non-Māori	Māori	Non-Māori	Māori
>20%	3.5 (5.2)	2.9 (4.1)	4.9 (7.3)	4.1 (5.8)
>15, ≤20%	2.4 (3.7)	2.1 (3.1)	3.4 (5.3)	3.0 (4.4)
>10, ≤15%	1.6 (2.6)	1.5 (2.2)	2.3 (3.6)	2.1 (3.1)
>5, ≤10%	0.8 (1.4)	0.9 (1.3)	1.2 (1.9)	1.2 (1.9)
>0, ≤5%	0.4 (0.7)	0.5 (0.7)	0.6 (1.0)	0.7 (1.0)

^*^Albeit with the reduction in adherence as in the base-case model of 22.5% over the five-year period of treatment.

In terms of monotherapy treatments, there was greater health gain in each risk strata for statin treatment than for anti-hypertensive treatment (eg, 34% higher for the second to lowest risk group [1030/771] Table S5 in the Supplementary Material). Similarly, for cost-effectiveness where treatment in all risk categories was cost-effective for only a statin (ICER range: $3740 to $43,500 (NZ$4140 to 48,100 in 2018)) but not in the lowest risk stratum for only an anti-hypertensive (ICER range: $6470 to $62,400 (NZ$7160 to 69,000 in 2018)).

## Discussion

### Main findings and interpretation

In the selected population group of middle-aged men aged 60–64, the potential offer of CVD preventive double therapy was highly cost-effective from a lifetime time horizon perspective at a 3% discount rate, for all levels of absolute risk. Nevertheless, in this case study we have performed this analysis for only one age-group of men and so we plan on further work to cover both sexes and for a much wider range of adult-age-groups; including among older age-groups where the benefits and risks of preventive pharmacotherapy may be more finely balanced.

The results of double therapy being cost-effective in all risk categories was not surprising given that these two medications (statins and anti-hypertensives) are effective, are relatively low cost, and previous international work on cost-effectiveness is favourable (see *Introduction*). The latter is especially the case for New Zealand, which has a central government agency (PHARMAC) that negotiates hard with the pharmaceutical industry for low prices – including for generics which all modelled statins and anti-hypertensives typically are.

Even so, there are likely to be even better value for money investments for preventing CVD such as advancing tobacco control, reducing sodium in processed foods, and modifying the obesogenic environment^2^. For example, our modelling of tobacco control interventions suggest that these are strongly cost-saving in New Zealand^12,14^, as are nearly all dietary salt reduction interventions (eg, in the processed food supply)^11,20,21^.

The individual level results in our study are not strictly comparable with other work. Even so, in a Dutch study of people with established CVD, the estimated per patient lifetime gain from taking a statin was 1.7 months (ie, 0.14 QALY at a 1.5% discount rate for a population with mean age of 61 years)^5^. From Australian work^6^, which did not specifically provide per capita results, we have estimated an average per person gain of taking a statin at 1.4 months (ie, for those with a ≥5% five year risk over their remaining life course, median age in late 60 s for men and early 70 s for women, and a discount rate of 3%).

### Study strengths and limitations

Strengths of this study included that it was built on a well-established original model (the TC-MSLT Model) that captured downward CVD incidence and case-fatality trends. The model also had a rich level of parameterisation with very detailed epidemiological and health cost data for New Zealand. The enhanced model had the benefits of using CVD risk data from a synthetic national population that used a New Zealand-specific CVD risk equation that took into account ethnicity (albeit an equation that has been further refined subsequently^27^). Furthermore, the CVD health problem being addressed is a major one for all high-income countries and it is well-defined in the sense that doctors regularly assess absolute CVD risk in their patients in the New Zealand setting, and low-cost preventive medications are available to prescribe. This work is also novel in terms of the level of modelling sophistication for determining health gain and cost-effectiveness within absolute risk strata.

Limitations that may mean we have underestimated health gains (and therefore underestimated cost-effectiveness), include assuming a future downward trend in CVD incidence and background mortality; this might not hold given the obesity epidemic (see *Introduction*). Further under-estimation may occur due to not including benefits around: preventing peripheral vascular disease and chronic kidney disease; from better controlling high blood pressure itself (eg, headaches from hypertension); and possibly the psychological reassurance or anxiety reduction provided by being on preventive medication. There is also some evidence that statins are associated with “lower risks of dementia and cognitive impairment, venous thrombo-embolism, fractures and pneumonia” – but also possibly increased risks of myopathy and diabetes^28^. However, in the systematic review by the US Preventive Services Task Force^29^, statins were not clearly associated with either myalgias (RR, 0.96 [95% CI, 0.79 to 1.16]), or increased risk of diabetes (RR, 1.05 [95% CI, 0.91 to 1.20]).

We may have also underestimated benefits for the higher risk strata and over-estimated those for the lowest risk strata. That is the benefit of statins appears to be probably disproportionately greater for those at highest risk given that those at higher risk will typically have higher cholesterol levels and there is evidence from a systematic review that statin treatment benefits will be greater for those with higher baseline cholesterol levels^30^.

Offsetting the above likely underestimation of health gains, is failure to adequately capture likely higher morbidity and mortality among those initially benefitting from treatment. In our modelling the fraction of the simulated cohort that has a CVD event prevented in the five years of treatment is assumed to have the same future CVD event rates as all surviving members of the cohort. Yet if people who have a CVD event prevented due to treatment have a higher risk of future CVD events than the remainder of people in the nominally same CVD risk strata (as seems plausible), then we will have overestimated survivorship and health gains. However, we are not aware of data on the magnitude of any such effect to allow its inclusion in the modelling.

Our base-case parameters used the best available New Zealand evidence (ie, 92% screened, 77% uptake of pharmacotherapy and an overall 22.5% decline in adherence over the five-year intervention period). But in some other countries the uptake and adherence may be improved with the current availability of fixed dose combinations that combine a statin and anti-hypertensive into a single tablet rather than two (which are not yet available in New Zealand^31^).

We also lacked adequate data to capture the potential adverse effects of preventive pharmacotherapy (eg, the possible association with muscle pain from statins as mentioned above) prior to discontinuation of treatment. That is, our model assumed those developing significant adverse effects became immediately non-adherent. Nevertheless, our model still did not capture the disutility for those who remain adherent but experience medication disutility simply from having to take daily medicine.

Finally, our results were only for men aged 60–64, yet the cost-effectiveness of the intervention will undoubtedly vary with different age groups, an area we are researching further. We would also be open to model comparison exercises with other research groups (ie, to evaluate model structure uncertainty) as has been successfully done with diabetes models via the Mount Hood Challenge process.

### Potential implications for future research

There is a need to extend this research to a wider range of adult age-groups (as referred to above), especially to the very elderly where harms from preventive medication may be greater. A more sophisticated analysis could compare the impact of packages of pharmacotherapy with various lifestyle interventions eg, smoking cessation, dietary changes (eg, sodium reduction), and increased physical activity.

### Potential implications for policy-makers

Policy-makers should consider these results alongside many other estimates for health gain, cost impacts and cost-effectiveness from CVD preventive interventions, as per an Australian and New Zealand online interactive league table^32^. System-level interventions for preventing CVD (eg, tobacco control, changing the obesogenic food environment etc) will typically have larger impact and be more likely to actually save costs. Nevertheless, offering double therapy as assessed in this study seems to be a cost-effective use of public health resources. Given that reducing health inequalities is also a health sector goal in many countries, then specifically promoting preventive pharmacotherapy for select population groups could be prioritised (eg, as per successful past New Zealand work on increasing statin use by Māori^10^).

In terms of more informed decision-making between patients and clinicians around taking preventive pharmacotherapy, our estimates around average extra months of quality-adjusted life gained (Table 6) are of potential value. These could be included in online tools that patients could use when making decisions around taking daily medication.

## Conclusions

In the selected population group of middle-aged men aged 60–64, the offer of CVD preventive double therapy (a statin and an anti-hypertensive) was highly cost-effective by conventional criteria for all levels of absolute CVD risk. Even so, more population-level interventions such as advancing tobacco control are more likely to generate large health gains and are more likely to be cost-saving. But at the individual level, patient considerations are critical as some people may decide that the average gain of 0.6 to 4.9 months of extra life (or less than a month with a five-year time horizon) does not justify the taking of daily medication.

## Supplementary information

Supplementary Material

## Data Availability

Supplemental information with additional methods and results is attached. Sharing of anonymised cohort data with other researchers or official agencies of the other epidemiological and costing data will generally be possible on request from the authors (pending approval of the relevant official agencies).
