# Supplementary material for: Preventive Pharmacotherapy for Cardiovascular Disease: A Modelling Study Considering Health Gain, Costs, and Cost-Effectiveness when Stratifying by Absolute Risk
Source: Sci Rep. 2019 Dec 20;9:19562. doi: 10.1038/s41598-019-55372-8 (PMC6925295; doi:10.1038/s41598-019-55372-8)
Supplement: Supplementary file 1 — Supplementary Material [file 41598_2019_55372_MOESM1_ESM.docx]

### Supplementary Material for: Preventive Pharmacotherapy for Cardiovascular Disease: A Modelling Study Considering Health Gain, Costs, and Cost-Effectiveness when Stratifying by Absolute Risk

Nhung Nghiem^1*^, Josh Knight^2 3^, Anja Mizdrak^1^, Tony Blakely^1 3^, Nick Wilson^1^

^1^ BODE^3^ Programme, University of Otago, Wellington, New Zealand

^2^ University of Auckland, Auckland, New Zealand

^3^ University of Melbourne, Melbourne, Australia

* Corresponding author: Dr Nghiem, Email: nhung.nghiem@otago.ac.nz

Figure S1: Calibration process framework for developing the CVD absolute risk model


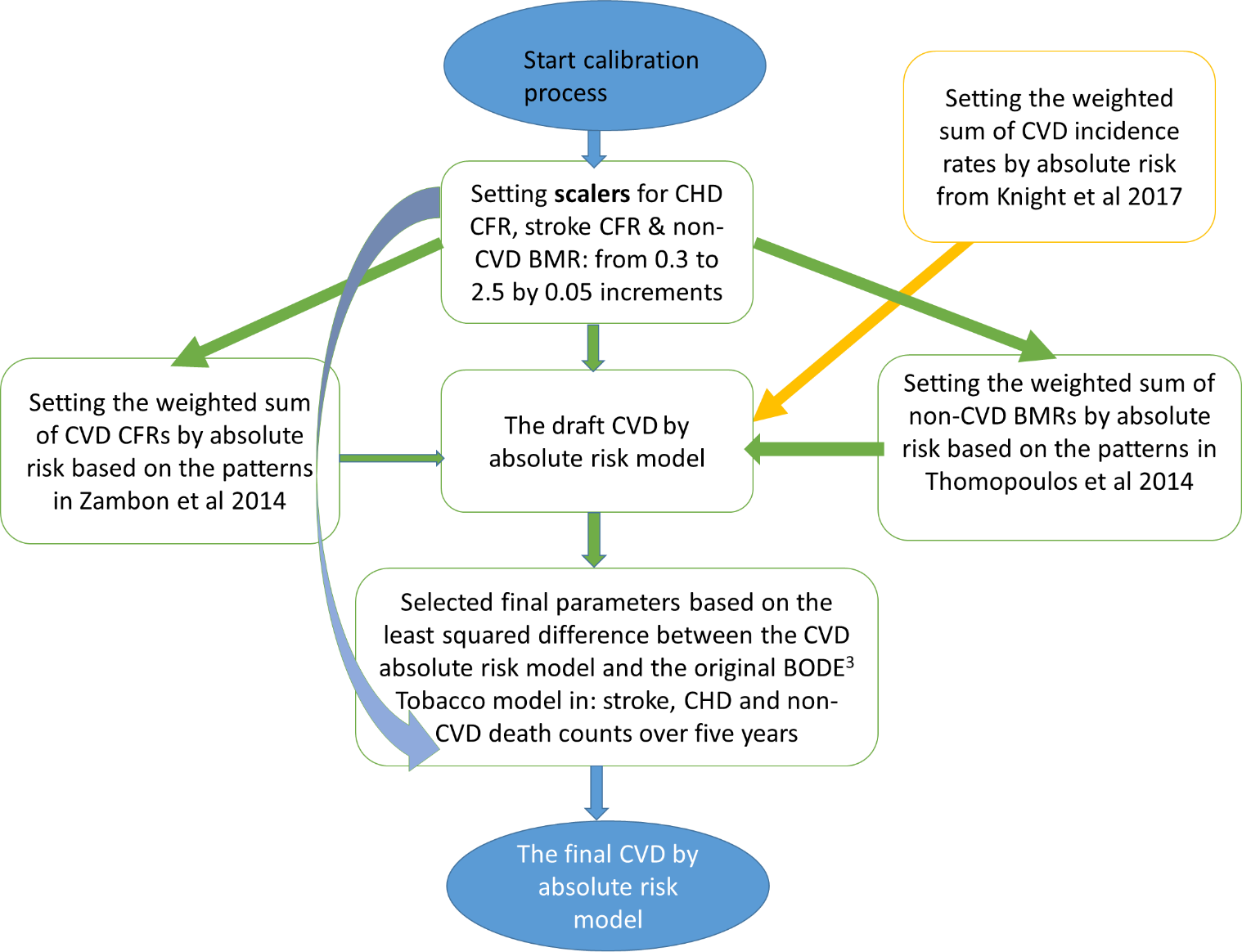


NB: We also checked the model by randomly drawing the scalers from 0 to 3, with 10,000 iterations; however, there was no further improvement in the min squared difference of the death counts.

Table S1: Full summary of epidemiological and cost parameters used in the modelling

| **Input parameter/s** | **Source** | **Derivation/ Application to Model/ Assumption** | **Heterogeneity** | **Expected Value and 95%UI** | **Distribution** |
| --- | --- | --- | --- | --- | --- |
| ***Background epidemiological parameters*** | | |  |  |  |
| Disease prevalence and incidence from the original TC-MSLT Model | See ^13^ | Data for CHD and stroke came from the original TC-MSLT Model. | Variation by Māori/non-Māori (also by age/sex in the original model) | See ^13^ | See ^13^ |
| Disease trends into the future from the original TC-MSLT Model | See ^13^ | As per the NZ Burden of Disease Study (NZBDS)^27^ we assumed a continued decline in incidence rates for both CHD and stroke of 2.0% annually, and also a 2.0% annual reduction in case fatality (ie, reflecting improved treatment and management). | Same trends applied to both groups | No uncertainty | No uncertainty |
| Disease morbidity rate per capita from the original TC-MSLT Model | See ^13^ | For each of the diseases, there was an assigned disability rate (DR; by sex and age) equal to YLDs for that disease (scaled down to adjust for comorbidities) from the 2006 NZBDS projected forward to 2011, divided by the disease prevalence (above). This DR was assigned to the proportion of the cohort in each disease state. No trend. | Nil (albeit variation by age/sex in the original model) | Uncertainty: ±10% SD. | Normal |
| Background mortality rates (when excluding CHD and stroke) | See ^13^ | Trends for all-cause mortality were made consistent with long-run mortality trends for NZ (annual 2.25% mortality decline for Māori and 1.75% per annum for non-Māori). Trends were modelled out to 2026, with no subsequent decline for both ethnic groupings thereafter. | Variation by Māori/non-Māori (and age/sex in the original model) | Nil uncertainty | Not applicable |
| Total morbidity per capita in 2011 from the original TC-MSLT Model | See ^13^ | The per capita rate of years of life lived with disability (YLD) was from the NZBDS by sex, age, and ethnicity. No trend (ie, assumed constant into the future, though further work on this issue is planned for the future). | Variation by Māori/non-Māori (and age/sex in the original model) | Uncertainty: ±10% SD | Log-normal |
| ***Intervention parameters*** | | |  |  |  |
| GP level screening for CVD risk: provision of offer and with GP asking at opportunistic consultations as a backup (for both double therapy and single medications) | National DHB data | National CVD assessment coverage data by DHB (and PHO) are available for the eligible population within a 5-year period (also for Māori and non-Māori CVD).^28^ Of note is the primary health organisations contributing to the PREDICT cohort study had risk assessed between 79% and 88% of their enrolled eligible patients.^29^ We assumed that GPs accurately assess CVD risk within the broad risk categories used in current clinical practice in NZ. | Variation by Māori/non-Māori | Māori: 86%  Non-Māori: 92%  (using median values for all DHBs, no mean values available) | Beta, SD= ±5% |
| Intervention uptake by patients when recommended by GP (for both double therapy and single medications) | NZ data^29^ | Use of CVD medications in NZ adults in the 55-64 year age-group who have had past CVD is 76.7% for blood pressure (BP)-lowering medications.^29^ It is even higher in the equivalent 85+ age group at 89.0%. We used the former value as a proxy for the starting level for those recommended to use CVD preventive pharmacotherapy by their GP (with other patients assumed to prefer lifestyle modifications or nothing).  **Heterogeneity:** Some differences exist in use of CVD medications by population group^30^ but we did not model this difference. Eg, for Māori vs non-Māori (64% vs 66%) and by age-group 55-64 year olds vs 65-74 year olds (68% vs 70%).^30^ Of note it is likely that these reported levels of use reflect both uptake of medication and adherence to it. | No variation (see details in the column to the left) | 77% overall | Normal, SD= ±10% |
| Decline in adherence to pharmacotherapy throughout the 5-year intervention period (for double therapy and single medications) | NZ data^31^ and authors assumptions | Data from the PREDICT cohort suggest that for those starting on both BP-lowering and lipid-lowering medications, the proportion using both declines to between 84% and 89% at the 3-year point (with trivial variation by CVD risk strata; data from the Appendix in Mehta et al).^31^ So we took the mid-point (86.5%) of this range and we assumed a linear decline of adherence of 4.5% percentage points per year – and continued this for the full 5-year period (to give an overall 22.5% decline).  **Heterogeneity:** See notes in the row above which may reflect both uptake of medication and adherence to it. Also of note are data on prescription dispensing (for those on both BP lowering and lipid-lowering medication and with a known history of CVD and with a relevant prescription history from their primary care provider. Differences were also small: men (94%), women (93%), Māori (91%), European (94%), 55-64 year olds (92%) and 65-74 year olds (95%).^30^ | No variation (see details in the column to the left) | Over the whole 5y period a 22.5% linear decline in adherence | Beta distribution (SD +/-5% of the cumulative reduction value) |
| Medication disutility / psychological reassurance from being on medication | Not applicable | We assumed that medication disutility was largely captured by both reduced uptake of medication and the reduction in adherence over time (as detailed above). That is, the non-uptake group includes those who don’t like taking medication or who consider themselves to be intolerant of medication. Similarly, the non-adherent group are likely to include those who develop adverse effects from medication. On the other hand we did not include psychological reassurance benefits from taking the medication. Similarly, we did not include potential adverse lifestyle changes from this reassurance (eg, consuming more unhealthy food due to the potential reassurance provided from taking statins – given evidence from a study involving taking polypills for CVD prevention change).^32^ | Not applicable | Not applicable | Not applicable |
| Effect of CVD preventive pharmacotherapy on risk of CHD and stroke events | See Table 4 | For the double therapy intervention, we assumed non-adherence resulted in full discontinuation of CVD preventive pharmacotherapy (not a shift to single medications). Similarly, for the single medications we assumed that when there was non-adherence, there was not switching to one of the other two single CVD preventive medications. | No variation | See Table 4 | Log-normal |
| ***Sensitivity and scenario analyses (for both double therapy and single medications)*** | | | | | |
| Varying the discount rate | We used 0% and 6% in sensitivity analyses (as per our BODE^3^ modelling protocol^26^). | | | | |
| Equity analysis | In this analysis we gave the Māori population the same potential envelope of health gain as per non-Māori, ie, the same morbidity and mortality rates as non-Māori.^33^ This prevents Māori in the analysis from effectively being penalised due to poorer existing health relative to non-Māori. | | | | |
| Halving of effect sizes for risk reduction | Authors assumptions | This scenario was considered given that the trial data might not be fully generalisable to the adult population in this target age-group (eg, trials tend to involve patients with elevated risk levels) | No variation | See values in Table 4 (all halved for each medication) | Log-normal |
| 5-year time horizon | As per the base-case analysis, but where the benefits (QALYs gained) and health costs were tallied up at 5 years. | | | | |
| 10-year time horizon | As per the base-case analysis, but where the benefits (QALYs gained) and health costs were tallied up at 10 years. | | | | |
| 20-year time horizon | As above but for the 20-year point. | | | | |
| Continuing use of therapy for 10 years (i.e. extending intervention duration in base model from 5 years to 10 years) | Authors assumptions informed by NZ data.^30^ | We assumed that after the initial 5-year decline in adherence, that adherence would then plateau (as per above in the 50% to 70% range). Of note is that for those in NZ with a known history of CVD, the use of two CVD medication categories (BP-lowering and lipid-lowering) was 70% in the older 65-74 year old age-group.^30^ | Nil (see above for adherence) |  |  |
| Continuing use of therapy for 20 years | As in the row above but for 20-years. | |  |  |  |
| ***Costs*** |  |  |  |  |  |
| Background health system costs for all citizens (adjusted for CHD and stroke costs) | As per BODE^3^ costing methods^34^ | Linked health data (hospitalisations, inpatient procedures, outpatients, pharmaceuticals, laboratories, and expected primary care usage) for each individual in NZ for the period 2006–2010 had unit costs assigned to each event, and then health system costs were estimated – adjusting for CHD and stroke costs (NZ$2011). No trend. | Nil | Uncertainty: ±10% SD. | Log-normal |
| GP visits, prescriptions, pharmaceutical costs | See Table 3 | See Table 3 | Nil | See Table 3 | See Table 3 |

Note: UI = uncertainty interval.

Table S2: Selected additional description of intervention-specific cost parameter details

| **Input Parameter** | **Source** | **Derivation/Application to Model/Assumption** | **Expected Value and 95%UI** | **Distri-bution** |
| --- | --- | --- | --- | --- |
| ***Costs for CVD assessment and being prescribed CVD preventive pharmacotherapy*** | | | | |
| GP visits for initial CVD risk assessment and on-going prescriptions and check-ups  (Same for double therapy and single medications) | PHARMAC cost resource manual^35^ | We assumed an average of two extra visits per year at $75 per visit (with two additional prescriptions a year being via telephone messaging – see below). This amount covers both patient co-payment and the government contribution. To this total of $150 we added 50% overhead cost as per the BODE^3^ Protocol, giving a total of $225 (or $218 in 2011 prices).  There is quite large uncertainty given that some visits that include a CVD management component might be combined with visits to the GP for other reasons. Also when people are considered to have stable BP/lipids they might only visit the GP once a year, but if these risk factors are unstable or they are getting adverse effects from medication, there might be several CVD-related visits per year. Also with increasing use of home BP monitoring this may trigger additional GP visits (for real BP increases or false positives due to declining device calibration). But alternatively, such home monitoring might also provide reassurance and result in fewer GP visits. A final limitation is that we did not assume a separate cost associated with capitation payments to the (minority) of general practices being paid in this way. | NZ$218 (in 2011 dollars) per annum over the five year intervention period | Gamma, SD ±20% |
| Fasting lipid test on first consultation (required for all CVD risk assessment, both double therapy and single medications) | HealthTracker data for 2011 | The test cost was $7.62. As HealthTracker data did not include a fee for taking the blood test, we used an administrative fee used in the commercial sector of $12 per test (<http://www.apath.co.nz/charges-lab-tests>). The total was $29.62 ($7.62 + $12) in 2017 prices, with this being $28.29 at year 2011 prices. | $28.29  (in first year of five year intervention period only) | Gamma, SD ±10% |
| Two annual prescriptions via telephone from GP (same for double therapy and single medications) | PHARMAC cost resource manual^35^ | In addition to the two extra doctor visits detailed above, we assumed two telephone calls per year to the general practice for obtaining prescriptions at $15 charge per batch in 2015 dollars as per the recommended PHARMAC costing (regardless of number of prescription items). | $28.93 per annum (2011 dollars) | Gamma, SD ±10% |
| Pharmacist payments for double therapy (2 medicines at 4 times year) | PHARMAC cost resource manual^35^ and pricing data for an agreement with community pharmacies^36^ | The total cost used (comprising the service fee, handling fee and the transition payment)  was $5.44 per prescription item. For 4 prescriptions per year this is $43.52 (that is $5.44 x 2 x 4 in 2017 dollars). Of note is that within the $5.44 amount the proportion that is patient co-payment may vary as some patients will meet the criteria for free dispensing (20 scripts per year) or have reduced fees from having a Community Services Card. | $41.57 per annum (2011 dollars) | Gamma, SD ±10% |
| Pharmacist payments for dispensing single medications (1 dispensing 4 times year) | As above | As above except for just one item per prescription ie, $21.76 (that is $5.44 x 4 = $21.76 in 2017 dollars). | $20.78 per annum | Gamma, SD ±10% |
| ***Pharmaceuticals*** |  |  |  |  |
| Lipid-lowering medication (same for double therapy and single medications) | PHARMAC Online Schedule in 2017 (<https://www.pharmac.govt.nz/Schedule>) | The official NZ guidelines we used,^37^ suggest starting with simvastatin 40 mg. There is uncertainty associated with the price due to the possibility that Pharmac made deals with the manufacturer (eg, around discounts or as part of package deals with other medicines). The 2017 price was $2.83 for 90 tablets (40mg each) = $11.48 per annum. | $10.97 (2011 dollars) | Gamma, SD ±10% |
| Anti-hypertensive (same for double therapy and single medications) | As above | The official NZ guidelines we used,^37^ didn’t mention a preferred medicine. We note one NZ study reported that in 2011 the most common BP lowering medications were ACE inhibitors (63%) and beta blockers (also 63%).^30^ So we used a typical ACE inhibitor: enalapril 20mg ($1.78 per 100, equivalent to $6.50 per annum). | $6.21 (2011 dollars) | Gamma, SD ±10% |
| Total annual cost of double therapy | See above | The medications detailed in the two preceding rows. | $17.18 per annum (2011 dollars) | See for individual medicines |

Table S3: Health gain (QALYs) and net health system cost impacts for all scenario and sensitivity analyses for the double therapy intervention in Māori and non-Māori men aged 60-64 years (showing expected values)

| **Five-year cumulative absolute risk strata** | **QALYs gained (non-Māori)** | **QALYs gained (Māori)** | **QALYs gained (ethnic groupings combined)** | **Net costs in NZ$ million (ethnic groupings combined)** | **ICER (NZ$ per QALY gained)** |
| --- | --- | --- | --- | --- | --- |
| ***Equity analysis*** |  |  |  |  |  |
| >20% | 16.7 | 11.3 | 27.9 | $0.04 | $1420 |
| >15, ≤20% | 55.3 | 31.8 | 87.2 | $0.16 | $1810 |
| >10, ≤15% | 262 | 102 | 364 | $1.16 | $3190 |
| >5, ≤10% | 1410 | 199 | 1610 | $14.7 | $9130 |
| >0, ≤5% | 1330 | 35.7 | 1370 | $34.3 | $25,100 |
| ***Intervention has half the effect size*** | | |  |  |  |
| >20% | 8.19 | 5.00 | 13.2 | $0.09 | $6770 |
| >15, ≤20% | 27.4 | 14.1 | 41.5 | $0.39 | $9310 |
| >10, ≤15% | 130 | 45.4 | 175 | $2.50 | $14,200 |
| >5, ≤10% | 702 | 88.8 | 791 | $23.7 | $30,000 |
| >0, ≤5% | 663 | 16.1 | 680 | $44.9 | $66,100 |
| ***Discount rate = 0%*** |  |  |  |  |  |
| >20% | 25.0 | 14.4 | 39.4 | $0.12 | $3130 |
| >15, ≤20% | 85.0 | 41.6 | 127 | $0.39 | $3110 |
| >10, ≤15% | 414 | 137 | 551 | $2.00 | $3640 |
| >5, ≤10% | 2320 | 276 | 2590 | $17.2 | $6620 |
| >0, ≤5% | 2250 | 51.4 | 2300 | $35.5 | $15,400 |
| ***Discount rate = 6%*** |  |  |  |  |  |
| >20% | 11.6 | 7.44 | 19.0 | -$0.001 | Dominant |
| >15, ≤20% | 37.8 | 20.5 | 58.3 | $0.05 | $936 |
| >10, ≤15% | 175 | 64.1 | 239 | $0.85 | $3530 |
| >5, ≤10% | 919 | 122 | 1040 | $14.0 | $13,500 |
| >0, ≤5% | 852 | 21.6 | 873 | $33.9 | $38,800 |
| ***Intervention runs for 20 years*** | | |  |  |  |
| >20% | 52.4 | 25.3 | 77.7 | $0.12 | $1560 |
| >15, ≤20% | 182 | 74.6 | 256 | $0.46 | $1800 |
| >10, ≤15% | 907 | 251 | 1160 | $3.27 | $2830 |
| >5, ≤10% | 5220 | 524 | 5750 | $40.6 | $7070 |
| >0, ≤5% | 5190 | 100 | 5290 | $96.4 | $18,300 |

Table S4: Health gain (QALYs) and net health system cost impacts for all scenario and sensitivity analyses from providing double therapy for different time periods into the future (all for men aged 60-64 years and focusing on expected values)

| **Five-year cumulative absolute risk strata and scenario** | **5 years into the future** | | **10 years into the future** | | **20 years into the future** | |
| --- | --- | --- | --- | --- | --- | --- |
|  | **Health gain (QALYs)** | **Net costs (NZ$ million)** | **Health gain (QALYs)** | **Net costs (NZ$ million)** | **Health gain (QALYs)** | **Net costs (NZ$ million)** |
| ***>20%*** |  |  |  |  |  |  |
| Base-case (for comparison) | 3.25 (2.52 to 4.01) | $-0.088 ($-0.168 to $-0.013) | 10.7 (8.35 to 13.0) | $-0.114 ($-0.201 to $-0.032) | 22.9 (17.6 to 28.0) | $-0.023 ($-0.092 to $0.042) |
| Equity analysis | 3.32 | -$0.09 | 11.1 | -$0.12 | 23.7 | -$0.02 |
| Intervention runs for 10 years | 3.34 | -$0.09 | 14.9 | -$0.22 | 41.4 | -$0.09 |
| Intervention runs for 20 years | 3.34 | -$0.09 | 15.0 | -$0.22 | 53.4 | -$0.20 |
| Half the effect size | 1.61 | $0.03 | 5.28 | $0.01 | 11.2 | $0.06 |
| 0% discount rate | 3.55 | -$0.10 | 12.8 | -$0.13 | 31.3 | $0.00 |
| 6% discount rate | 2.98 | -$0.08 | 9.0 | -$0.10 | 17.0 | -$0.04 |
| ***>15, ≤20%*** |  |  |  |  |  |  |
| Base-case (for comparison) | 9.6 (7.43 to 11.8) | $-0.112 ($-0.385 to $0.149) | 30.8 (24.3 to 37.4) | $-0.254 ($-0.568 to $0.036) | 68.2 (54.1 to 83.1) | $-0.067 ($-0.341 to $0.195) |
| Equity analysis | 9.7 | -$0.11 | 31.6 | -$0.25 | 70.5 | -$0.06 |
| Intervention runs for 10 years | 9.8 | -$0.12 | 43.5 | -$0.48 | 125 | -$0.28 |
| Intervention runs for 20 years | 9.8 | -$0.12 | 43.8 | -$0.49 | 163 | -$0.59 |
| Half the effect size | 4.72 | $0.25 | 15.2 | $0.18 | 33.7 | $0.28 |
| 0% discount rate | 10.4 | -$0.12 | 36.6 | -$0.30 | 93.9 | -$0.03 |
| 6% discount rate | 8.74 | -$0.10 | 25.9 | -$0.21 | 50.5 | -$0.07 |
| ***>10, ≤15%*** |  |  |  |  |  |  |
| Base-case (for comparison) | 37.6 (29.1 to 47.1) | $0.736 ($-0.582 to $2.123) | 118 (92.8 to 144) | $-0.103 ($-1.636 to $1.436) | 270 (211 to 328) | $0.18 ($-1.32 to $1.7) |
| Equity analysis | 37.9 | $0.73 | 120 | -$0.11 | 276 | $0.17 |
| Intervention runs for 10 years | 38.8 | $0.69 | 171 | -$0.24 | 502 | -$0.53 |
| Intervention runs for 20 years | 38.8 | $0.69 | 172 | -$0.27 | 669 | -$1.21 |
| Half the effect size | 18.7 | $2.28 | 58.6 | $1.86 | 134 | $2.00 |
| 0% discount rate | 40.9 | $0.75 | 140 | -$0.30 | 373 | $0.06 |
| 6% discount rate | 34.5 | $0.71 | 100 | $0.04 | 199 | $0.28 |
| ***>5, ≤10%*** |  |  |  |  |  |  |
| Base-case (for comparison) | 162 (122 to 208) | $18.2 ($9.2 to $28.5) | 483 (371 to 599) | $12.95 ($3 to $23.6) | 1110 (864 to 1350) | $10.7 ($0.3 to $21.8) |
| Equity analysis | 162 | $18.09 | 484 | $12.86 | 1120 | $10.64 |
| Intervention runs for 10 years | 168 | $17.86 | 720 | $23.50 | 2110 | $14.45 |
| Intervention runs for 20 years | 168 | $17.86 | 726 | $23.27 | 2900 | $23.95 |
| Half the effect size | 80.5 | $25.41 | 240 | $22.82 | 550 | $21.68 |
| 0% discount rate | 176 | $19.05 | 571 | $12.55 | 1,530 | $8.95 |
| 6% discount rate | 148 | $17.23 | 408 | $12.95 | 814 | $11.73 |
| ***>0%, ≤5%*** |  |  |  |  |  |  |
| Base-case (for comparison) | 141 (108 to 182) | $41.7 ($26.9 to $58.6) | 399 (311 to 504) | $36.2 ($21 to $53.6) | 890 (701 to 1100) | $31.7 ($15.8 to $49.1) |
| Equity analysis | 141 | $41.91 | 399 | $36.41 | 891 | $31.91 |
| Intervention runs for 10 years | 147 | $41.73 | 613 | $66.36 | 1740 | $53.18 |
| Intervention runs for 20 years | 147 | $41.73 | 619 | $66.36 | 2460 | $89.09 |
| Half the effect size | 70.3 | $48.64 | 199 | $45.91 | 444 | $43.73 |
| 0% discount rate | 153 | $44.27 | 472 | $37.45 | 1230 | $30.50 |
| 6% discount rate | 130 | $39.73 | 339 | $35.27 | 659 | $32.45 |

Table S6: Impact of only a statin or only an anti-hypertensive on health gains and net health system cost-savings for 60-64-year-old men (Māori and non-Māori combined) over their remaining life-course from the offer of screening*

| **Five-year cumulative absolute risk strata** | **Only a statin** | | | **Only an anti-hypertensive** | | |
| --- | --- | --- | --- | --- | --- | --- |
|  | **QALYs gained** | **Net costs (NZ$ million)** | **ICER (NZ$ per QALY gained)** | **QALYs gained** | **Net costs (NZ$ million)** | **ICER (NZ$ per QALY gained)** |
| >20% | 17.3 (12.0 to 22.4) | $0.062 ($0.014 to $0.114) | $3740 ($711 to $7900) | 12.8 (8.55 to 17.2) | $0.078 ($0.034 to $0.129) | $6470 ($2280 to $12,900) |
| >15, ≤20% | 54.1 (37.5 to 70.6) | $0.263 ($0.06 to $0.477) | $5110 ($1000 to $10,900) | 40.4 (26.6 to 54.5) | $0.335 ($0.151 to $0.541) | $8850 ($3390 to $18,400) |
| >10, ≤15% | 229 (160 to 301) | $1.736 ($0.518 to $3.064) | $7950 ($1900 to $16,100) | 169 (116 to 228) | $2.16 ($1.06 to $3.47) | $13,400 ($5600 to $25,900) |
| >5, ≤10% | 1030 (728 to 1320) | $18.36 ($9.18 to $29.05) | $18,700 ($7700 to $34,200) | 771 (529 to 1010) | $20.8 ($12 to $31.7) | $28,200 ($13,500 to $52,000) |
| >0, ≤5% | 883 (615 to 1140) | $36.9 ($21.9 to $54.5) | $43,500 ($22,400 to $73,700) | 663 (453 to 865) | $39.8 ($25.5 to $57.3) | $62,400 ($33,600 to $104,000) |

* For those starting with no past CVD events and no past CVD medication; using 92% screened, 77% uptake and an overall 22.5% decline in adherence over time (the same for both medications), for a 5-year treatment period only, 3% discount rate.
